# Supplementary material for: Assessing the drivers of syphilis among men who have sex with men in Switzerland reveals a key impact of screening frequency: A modelling study
Source: PLoS Comput Biol. 2021 Oct 26;17(10):e1009529. doi: 10.1371/journal.pcbi.1009529 (PMC8570495; doi:10.1371/journal.pcbi.1009529)
Supplement: Attachment — Submitted filename: Response_to_reviewer_with_supp.pdf [file pcbi.1009529.s002.pdf]

Dear editors,

We are submitting the revised manuscript along with this point-by-point response to the reviewers' comments. Reviewers' comments are reproduced in bold and changes to the manuscript in blue font along this document.

We thank the editors for the opportunity to revise, and the reviewers for their comments and suggestions, which we fully addressed in this revision.

Gratefully,

Suraj Balakrishna on behalf of all co-authors.

Reviewer #1:

1. The introduction is very shallow. The authors should do more by reviewing existing literature in this area.

Response: We revised the introduction section to the light of further existing literature. It now reads.

'Syphilis, a sexually transmitted infection caused by bacteria *Treponema pallidum,* remains a major public health problem. Over the last decade, a rising number of diagnoses especially in men-who-have-sex-with-men (MSM) was observed in Western Europe and Northern America[1–3]. In Switzerland, the number of syphilis diagnoses in MSM tripled between 2006 and 2017[4]. A similar trend was observed for the incidence rate of syphilis in HIV-diagnosed MSM in the Swiss HIV Cohort Study (SHCS)[5–7]. Syphilis and HIV coinfections are common, and syphilis has been associated with increased risk or HIV transmission[8]. Understanding syphilis transmission dynamics while considering the role of HIV infection could provide insights towards more effective prevention efforts to reduce the incidence of syphilis.

The syphilis incidence in a population is largely determined by parameters such as the transmission probability per sexual partnership, the average rate of acquisition of new sexual partners and the duration of infectiousness, as well as by the prevalence of practices such as -oral sex, condomless anal intercourse, multiple sexual partnerships, and anal

intercourse with non-steady partners [9,10]. Prevention strategies such as the promotion condom use, risk-reduction counselling, and syphilis screening and treatment, have the potential to influence syphilis rates by modifying these key factors [11]. Recent studies show a decrease in condom use for anal sex in MSM with HIV diagnosis[11,12]. This situation may have caused the increased incidence of syphilis.

The natural course of syphilis has four stages: primary, secondary, latent, and tertiary. Almost all syphilis transmissions occur through direct skin-to-skin contact with active lesions during the primary and secondary stages and the disease remains transmissible for an average period of two to three months[13]. Studies have estimated the attack rate of syphilis within 30 days of exposure to sexual transmission of syphilis to be between 16 and 30%[14,15]. During the latent stage of syphilis, there are no clinical signs or symptoms of syphilis. If left untreated, the infection may reach its tertiary stage and may affect different organ systems. Hence it is essential to diagnose and treat people infected with syphilis as early as possible. A stepped wedge cluster randomized controlled trial showed that the routinized syphilis screening among men living with HIV increased the syphilis diagnosis[16]. Previous modelling studies suggest that more frequent syphilis screening could reduce incidence[17–20].

We aimed to identify possible drivers of the ongoing syphilis epidemic among MSM in Switzerland using a mathematical model (Figure 1) and to characterise the role of behavioral predictors (proxies for transmission risk of syphilis) that could best explain the changes in syphilis incidence observed over the last decade. Based on this model, we evaluated the effect of different screening strategies on syphilis incidence.'

Newly added citations in the introduction:

8. Wu MY, Gong HZ, Hu KR, Zheng H-Y, Wan X, Li J. Effect of syphilis infection on HIV acquisition: a systematic review and meta-analysis. Sex Transm Infect. 2020; sextrans-2020-054706. doi:10.1136/sextrans-2020-054706

11. Stoltey JE, Cohen SE. Syphilis transmission: a review of the current evidence. Sex Health. 2015;12: 103–109. doi:10.1071/SH14174
14. Moore MB, Price EV, Knox JM, Elgin LW. Epidemiologic treatment of contacts to infectious syphilis. Public Health Rep. 1963;78: 966–970.
15. Schroeter AL, Turner RH, Lucas JB, Brown WJ. Therapy for incubating syphilis. Effectiveness of gonorrhea treatment. JAMA. 1971;218: 711–713.
16. Burchell AN, Tan DHS, Grewal R, MacPherson PA, Walmsley S, Rachlis A, et al. Routinized Syphilis Screening Among Men Living With Human Immunodeficiency Virus: A Stepped Wedge Cluster Randomized Controlled Trial. Clin Infect Dis. 2021 [cited 13 Aug 2021]. doi:10.1093/cid/ciab582
17. Gray RT, Hoare A, Prestage GP, Donovan B, Kaldor JM, Wilson DP. Frequent testing of highly sexually active gay men is required to control syphilis. Sex Transm Dis. 2010;37: 298–305. doi:10.1097/OLQ.0b013e3181ca3c0a
18. Tuite AR, Fisman DN, Mishra S. Screen more or screen more often? Using mathematical models to inform syphilis control strategies. BMC Public Health. 2013. doi:10.1186/1471-2458-13-606
19. Saad-Roy CM, Shuai Z, van den Driessche P. A mathematical model of syphilis transmission in an MSM population. Math Biosci. 2016;277: 59–70. doi:10.1016/j.mbs.2016.03.017
20. Iboi E, Okuonghae D. Population dynamics of a mathematical model for syphilis. Appl Math Model. 2016;40: 3573–3590. doi:10.1016/j.apm.2015.09.090

2. **The objective of this study must be clearly stated from the onset**

Response: The objective of this study was stated in the last paragraph of the introduction of the originally submitted manuscript. We have slightly revised this paragraph to make ever clearer. It now reads:

‘We aimed to identify possible drivers of the ongoing syphilis epidemic among MSM in Switzerland using a mathematical model (Figure 1) and to characterise the role of behavioral predictors (proxies for transmission risk of syphilis) that could best explain the changes in syphilis incidence observed over the last decade. Based on this model, we evaluated the effect of different screening strategies on syphilis incidence.’

Also, in line with the reviewer’s suggestion, we have in addition modified the first sentence of the abstract in the methods section to clearly state the objective of this study from the onset. It now reads:

‘In order to identify the drivers of syphilis transmission in MSM in Switzerland between 2006 and 2017 as well as the effect of potential interventions, we set up an epidemiological model stratified by syphilis stage, HIV-diagnosis, and behavioral factors to account for syphilis infectiousness and risk for transmission.’

3. The authors talked about system of nonlinear equations for the study. This I could not find. Authors must include the model equations.

Response: The model equations were given in the supplementary section 2 and referenced in the methods section already in the originally submitted manuscript (last sentence of first paragraph of ‘Model of syphilis transmission’ subsection of methods section):

‘A complete description of the model, including model equations, is available in supplementary sections 1 and 2.’

We now refer to the section containing the model equations also in the first sentence of the corresponding subsection. It now reads:

‘To model the syphilis epidemic in MSM in Switzerland between 2006 and 2017, we set up a system of 32 coupled ordinary differential equations (See supplementary section 2 for the model equations).’

4. The results of the study must relate with previous literature and authors must state how this model or result improved on previous results.

Response: We have revised the existing paragraph in the manuscript that indicates previous literature and states how this model improved on such previous results to the light of further existing literature. The paragraph is now as follows:

‘Our transmission model enabled us to integrate data from different sources such as published data from the FOPH and the literature, and the results estimated from the SHCS and cross-sectional studies to assess the drivers of the syphilis epidemic in Switzerland. Previous studies have shown the potential impact of more frequent screening in MSM for syphilis on reducing syphilis infections[17–20]. An individual-based mathematical model that

simulates the formation and breakup of sexual partnerships and tracks the transmission of syphilis within a synthetic population of sexually active gay men showed that increasing the frequency of syphilis screening can have a large impact on reducing syphilis prevalence[17]. The study also emphasized targeted frequent screening of gay men who have large numbers of partners or who engage in group sex to be a more efficient way of reducing syphilis transmission. An agent-based, network model of syphilis transmission, representing a core population of 2000 Canadian MSM at particular high risk for syphilis, explicitly estimated the effect of different screening frequencies on the projected annual rates of syphilis in this population[18]. The study showed that increased syphilis screening frequency was consistently more effective than improved coverage for screening. Our model differs from these studies, in that we not only focused on a core group of MSM by means of transmission risk of syphilis but also stratified the model based on HIV status. To the best of our knowledge, this is the first study to link the syphilis epidemic in MSM_{wHD} with that in $MSM_{w/oHD}$ and to estimate the effect of screening strategies implemented in target groups of the population on the overall epidemic.'

Furthermore, our results strengthen the existing clinical guidelines as indicated in the originally submitted manuscript manuscript:

'Our study results thus robustly indicate that frequent screening of MSM with HIV diagnosis and with nsP is required to curb syphilis transmission. This supports the EACS guidelines which recommend to "consider more frequent than annual screening if at risk"[35].'

5. The fonts of the figures must be increased

Response: We have increased the fonts of all figures in the manuscript as much as possible. For figure 1, we could only increase the font size of to a limited extent due to four subfigures. However, the purpose of figure 1 is to illustrate the overview of the methods. As an alternative, we have referenced the corresponding figure in the subfigures of figure 1.

6. The authors should go through the entire work to check for typo errors

Response: We followed the reviewer suggestion and corrected for all typos we found after additional proofreading by co-authors and a native English speaker not involved in the study.

Reviewer #2: Though I've spent substantial time with the main manuscript and supplemental materials for the paper titled "Assessing the drivers of syphilis among MSM in Switzerland reveals a key impact of testing frequency: a modelling study", my understanding of this work is still incomplete. The authors are using a fairly complicated ODE model involving syphilis transmission, HIV demographics, heterogenous sexual activity classes, and history of syphilis infection. They use multiple sources of data for both parameterization and model fitting. They assess a few counterfactual intervention scenarios and conclude that increased screening among HIV+ MSM who report non-steady partners would be most effective, compared to increased screening of other groups. The work suggests that if syphilis screening among MSM with HIV were quadrupled (from once every other year to twice per year), syphilis incidence would drop to 0.04 per year from 4.91 per year—a drastic reduction that appears to lead to syphilis elimination in this modeled population, though this is not stated directly.
My main concerns involve elements of the methods and supplemental sections related to a) model construction, b) model parameterization, and c) model communication. The communication issues are the most serious, as I'm unable to assess whether critical model features are implemented in a suitable way.

Model construction:

1) Line 152 refers to 36 equations. Subsequent text (152-162) and Figure 1a would indicate only 32 equations (4 syphilis states, 2 HIV states, 2 nSP states, 2 syph_history states). What do the 4 remaining equations refer to?

Response: We apologize for this error. As this reviewer correctly pointed out, there are only 32 model equations. We corrected this in the revised manuscript.

2) HIV prevalence was not stable between 2006-2018 (line 236 and Supplemental Table 2): the prevalence grew from 3.5% in 2006 to 5.2% in 2018). It seems the serosorting parameters are problematic, as the probability of a contact being with an HIV+/- person will in part depend on the changing density of HIV+/- people. Otherwise, there is changing preference for HIV+ partners over the period as the prevalence changes.

Response: Thanks for raising this important point. As the reviewer pointed out, we have assumed constant serosorting probabilities for MSM with and without

HIV-diagnosis between 2006 and 2018 despite the changing prevalence of HIV. These probabilities were derived based on longitudinal data from Voluntary Counselling and Testing (VCT) centres, which suggested relatively time-independent serosorting contact probabilities over the study period. The scope for generalisation of these data is however limited since VCTs do not collect data as a clinical study/cohort would.

Regarding the SHCS, despite the changing HIV prevalence, the proportion of stable partners of same serostatus (HIV-positive partner) among MSM enrolled in the cohort has remained constant over time. While this suggests stable serosorting probabilities during the study period, we acknowledge that the scope of this finding is limited by the lack of explicit evidence on the HIV-status of occasional partners.

Thus, even though the available data is limited, it overall supports our choice of constant serosorting probabilities.

3) Critical: Tau (the relative risk due to reported non steady partners) does not appear to be described in the supplemental section 3.3. This seems like a critical parameter that is specified (i.e., not free). Can this be thought of as a contact rate inflation factor?

Response: We apologize for this omission. As the reviewer infers, tau can be thought of as a contact rate inflation factor. To further clarify this, we have included a paragraph on tau in the supplementary section 3.3:

'Tau is the relative risk due to reported non-steady partners (nsP). We found a significant association between reported nsP and incidence rate of syphilis in MSM diagnosed with HIV by using univariable marginal regression models, an extension of Cox-proportional hazards models, like the ones we performed in Roth *et al.,* 2020 (hazard ratio, HR = 3.167; 95% confidence interval, CI = 2.721-3.686). For simplicity, we assumed this HR as the relative risk due to nsP while calculating the force of infection. Tau could be thought as a contact rate inflation factor. In other words, MSM with nsP have a higher force of infection due to higher risk of having nsP than those without nsP.'

Model parameterization:
4) The data specified in supplemental table 2 all seem like point estimates with no variability. Is this wise? For example, do we really know the

serosorting probability with exact precision? Perhaps this should be varied. I understand you don't want to have an infinite number of "free parameters", but perhaps having partially informed group would be a good idea. Your inferences might be quite reliant on the choice of values for this serosorting parameter (and others).

Results: Thank you for raising this concern. As correctly pointed out, the number of possible 'free parameters' is limited by multicollinearity leading to identifiability issues. We fully agree that our inferences might be reliant on choice of values. Hence, we performed an additional sensitivity analysis where we sampled 50 sets of fixed parameters from given bounds (see supplementary table 5) around the point estimates using a Latin hypercube sampling algorithm. Sampling was followed by model optimization, and counterfactual scenario analyses. We have explained the results of this analysis and their implications for our finding in detail in the supplementary section 11*. We also refer to them in the revised manuscript as follows:

In 'Sensitivity analysis' subsection of methods section:

"Finally, we tested the robustness of our model results to fixed parameter sets by sampling 50 sets of fixed parameters from given bounds around the point estimates using a Latin hypercube sampling algorithm. Sampling was followed by model optimization, and counterfactual scenario analyses."

In the last paragraph of 'Sensitivity analysis' subsection of results section:

"Finally, we tested the robustness of our model by assessing if the model results were reliant on the choice of value of fixed parameters by sampling fixed parameter sets using Latin hypercube sampling followed by model optimization, and counterfactual scenario analyses (Supplementary section 11). We found that our model results were robust to the choice of fixed parameter sets."

*Please note that this entire section is new. Given its length, we have chosen to include supplementary section 11 at the end of this point-by-point reply.

Model communication:
5) Critical: Screening versus testing versus diagnosis. The supplementals give "diagnosis rates" in Table 2. The primary counterfactual intervention is increased screening. This language needs to be harmonized throughout.

There are subtle differences between rates of screening, testing, and diagnosis.

Response: Thanks a lot for raising this crucial point. We now consistently use the term ‘screening’ and omitted the term ‘testing’ throughout the revised manuscript. Furthermore, in supplementary table 2, we give the rate of transition from infected state to diagnosis state as $\Delta_{ID}^{v,r}$ (rate of becoming diagnosed from infected). Similarly, we define $\Delta_{LD}^{v,r}$ (rate of becoming diagnosed from non-infectious). As you pointed out, these are not ‘screening rates’. To clarify this, we have now included a paragraph in the supplementary section 3.4 explaining the relationship between ‘screening rates’ and ‘diagnosis rates’, and the rationale in deriving this relationship.*

*Given the length of the supplementary section 3.4, we have chosen to include at the end of this point-by-point reply.

6) As a reviewer it is critical for me to understand how screening and testing work in the model, as this is the primary intervention being proposed. From Section 3.4 in the supplemental; I’m trying to understand delta_LD and delta_ID, but I am confused. From the section text: “the rates of becoming diagnosed from infected and non-infectious are given by delta_LD and delta_ID respectively.” Does LD refer to latent disease? If so, the order is wrong in the above sentence.

Response: We apologize for misplaced order. This sentence has been corrected to:

‘The rates of becoming diagnosed from infected and non-infectious are given by delta_ID and delta_LD respectively.’

7) Again, in Section 3.4 of the supplemental, the text goes on to talk about 80% being infectious syphilis and the remaining “10%” being non-infectious; but what about the 10% leftover? I do not understand the remaining text in this section.

Response: We are sorry for this error. It has been changed to ‘20%’ being non-infectious.

8) Remaining in Section 3.4, I question the calculations, that they might require equilibrium conditions in order to apply the proportions as they do.

Response: As you pointed out, due to limited evidence, it would be hard to establish an equilibrium state i.e., the proportion of individuals in infectious state and non-infectious state to be constant throughout the study period. Based on the FOPH notification available for the period 2017 to 2019, the proportion of being diagnosed in infected and infectious state (I) is about 77 – 82%. Thus, to assess the robustness of our model, we performed additional sensitivity analysis that considered an interval around the estimated 'rate of becoming diagnosed from infected ($\Delta_{ID}^{v,r}$)'. We found that our model results were robust to the choice of $\Delta_{ID}^{v,r}$ even at near-equilibrium conditions. Please see supplementary section 11 for a detailed description. We refer to these findings in the revised manuscript as explained before (please see response to question 4).

9) I would appreciate how these rates might relate to measurable phenomena such as a routine screening rate that everyone might experience; people with symptoms (and recent infection acquisition) would then have an additional pressure to get tested due to either symptoms or contact tracing of an infected partner.

Response: We have now explained this in detail in section 3.4 of the revised manuscript, where we explain the relationship between 'diagnosis rates' and 'screening rates'. Please also see our response to question 5* to reviewer #2.

*Given the length of the supplementary section 3.4, we have chosen to include at the end of this point-by-point reply.

Remaining questions:
10 How does the model deal with international travel of Swiss MSM and foreign MSM travelling to Switzerland? Can the authors weigh in on the importance of imported syphilis? Might the SHCS have data related to partner acquisition while abroad, or sex with non-Swiss while in Switzerland? ~400 syphilis diagnoses is not very many, and I wonder whether the syphilis dynamics in Switzerland might be driven more by exogeneous factors than endogenous ones.

Response: Our model assumes the incidence rate of syphilis outside Switzerland to equal that in Switzerland. Cases are imported into Switzerland based on the migration rate. We chose to use this simplification for the following reasons:
As per the FOPH notification data on syphilis, the overall incidence rate of syphilis in Switzerland in 2016 was 8.7 cases per 100,000 population. This rate is comparable to many European countries. Based on the ECDC surveillance report 2016, incidence rate of syphilis in 28 EU/EEA Member States was 6.1 cases 100,000 population. This varies by country. For example, Germany and Italy, two countries with dense travelling networks with Switzerland, have 8.7 and 2.3 cases per 100,000 population, respectively. This indicates substantial diversity across countries. The incidence trends inside and outside Switzerland are likely to be similar (as consequence of direct transmission and concordant behavioural shifts). This would, in turn, result in collinearities complicating the estimation of external forces of infection.

Furthermore, according to the FOPH notification data on syphilis, the country of infection was known in 76.8% of the syphilis infections in MSM in 2017. Most of these infections (81.6%) occurred in Switzerland. This suggests that the syphilis epidemic in Switzerland is most likely driven by endogenous factors rather than exogeneous factors.

11) The supplemental materials indicate correlation between Beta and Beta_hiv, and initSyph_h1 and initSyph_h0. This looks like a potential identifiability issue. Are the findings related to counterfactual intervention effectiveness uniform across these dimensions? More explicitly, is targeting screening to HIV+ men still recommended across this spectrum?

Response: Thanks for raising your concerns on identifiability. As shown in figure S7, none of the collinearity indices (pairwise as well as all free parameters) included in the model have high enough collinearity or multicollinearity to hinder identifiability. Hence, our findings related to counterfactual intervention effectiveness are expected to hold true across these dimensions. Thus, targeted screening of MSM with HIV diagnosis would be recommended by the model despite the correlation between Beta and Beta_hiv, and initSyph_h1 and initSyph_h0.

12) Might HIV+ MSM enrolled in the SWHD study be different from HIV+ MSM not enrolled in the study? In particular, might they have a much higher

routine screening and diagnosis rate? Additionally, might their screening rate be more difficult to modify?

Response: We assume you mean the 'Swiss HIV Cohort Study (SHCS)'.
In this model, we defined stratification by HIV status as HIV-diagnosed and HIV-undiagnosed MSM. In Switzerland, >95% of MSM with HIV diagnosis are retained in care [Kohler *et al.,* 2015]. Hence, they are likely to have similar screening and diagnosis rates as observed in the SHCS. For the same reasons, we believe it should be feasible to modify the screening rates of MSM with HIV diagnosis not enrolled in the SHCS, for example by means of public health policies or campaigns.

13) The main conclusions would be strengthened if a measure of intervention efficiency were introduced; there are different numbers of tests being administered to the HIV+/- and behavioral class groups, and increased screening among HIV+ MSM who report non-steady partners might look even better given that this is probably not a large group of people.

Response: We thank the reviewer for this suggestion. We have now included a measure of intervention efficiency in the results of counterfactual scenario. It now reads:

"Secondly, we considered the impact of change in screening frequency for syphilis between the years 2006 and 2018. Increasing the frequency of screening for syphilis among MSM with HIV diagnosis and with nsP from once per year to twice per year during the observation period (corresponding to 48,576 syphilis screening tests instead of 24,179 tests), we estimated a reduction of 99.19% (0.04 vs 4.91 cases per 1000 person-years) in incidence rate of syphilis in 2017 compared to incidence rate obtained by the model fit in 2017. By contrast, we estimated only a reduction of 12.75% (4.28 vs 4.91 cases per 1000 person-years) in incidence rate of syphilis in 2017 by increasing the frequency of screening for syphilis among MSM without HIV diagnosis and with nsP from once per two years to twice per year during the observation period (corresponding to 1,289,070 syphilis screening tests instead of 332,226 tests)".

3.4 Rate of becoming Diagnosed

MSM can be diagnosed with syphilis when they are infected with syphilis and are in either an infected state (infectious) or a non-infectious state. The rates of becoming diagnosed from infected and non-infectious are given by Δ_{ID} and Δ_{LD} respectively. MSM in the non-infectious state (L) are usually asymptomatic and could only be diagnosed with syphilis due to routine screening and/or by partner notification. By contrast, those in an infected state (I) could be diagnosed with syphilis due to routine screening, partner notification and/or screening for syphilis due to symptoms. Furthermore, MSM in the non-infectious state (L) are less likely to be diagnosed with partner notification compared to those in the infected state (I) as the partners in the last 3 months period could be tracked easily compared to partners before 3 months. Thus, for simplicity, we assume that the diagnosis of syphilis in MSM in L state would only occur due to routine screening.

Thus, we derive $\Delta_{LD}^{v,r}$ (rate of becoming diagnosed from non-infectious) as the inverse of average time to routine screening ($\Delta_{scr}^{v,r}$).

$$\Delta_{LD}^{v,r} = \Delta_{scr}^{v,r}$$

MSM with HIV diagnosis are routinely screened for syphilis once a year. Hence, We assume that the average time to routine screening for MSM with HIV diagnosis to be 1 year. Although the recommendation of syphilis screening in MSM without HIV diagnosis is once per year, not everyone is routinely tested every year. Based on the European Men-Who-Have-Sex-With-Men Internet Survey (EMIS-2017), only 50% of the participants had syphilis screening in the past year[4, 5]. Hence, we assumed the average time to diagnosis in MSM without HIV diagnosis to be 2 years. The corresponding rates of switching from non-infectious to diagnosed for MSM with and without HIV diagnosis are 1 and 0.5 $year^{-1}$ respectively.

$$\begin{aligned} \text{i.e., } \Delta_{LD}^{1,r} = \Delta_{scr}^{1,r} &= 1 \\ \Delta_{LD}^{0,r} = \Delta_{scr}^{0,r} &= 0.5 \end{aligned}$$

Whereas, MSM in the infected state (I) who are symptomatic could be diagnosed with syphilis due to their symptoms, routine screening, or partner notification. Thus, we further assume that $\Delta_{ID}^{v,r}$ (rate of becoming diagnosed from infectious) is an additive effect of routine screening rates ($\Delta_{scr}^{v,r}$), due to symptoms ($\Delta_{sym}^{v,r}$), and partner notifications ($\Delta_{part}^{v,r}$).

$$\begin{aligned} \Delta_{ID}^{v,r} &= \Delta_{scr}^{v,r} + \Delta_{sym}^{v,r} + \Delta_{part}^{v,r} \\ \text{or, } \Delta_{ID}^{v,r} &= \Delta_{scr}^{v,r} + \Delta_{sp}^{v,r} \\ \text{where,} & \\ \Delta_{sp}^{v,r} &= \Delta_{sym}^{v,r} + \Delta_{part}^{v,r} \end{aligned}$$

Therefore, if we increase the routine screening frequency by a factor of x, rates of becoming diagnosed from non-infectious and infected would be given by:

$$\Delta_{LD}^{v,r} = \Delta_{scr}^{v,r} * x$$

$$\Delta_{ID}^{v,r} = \Delta_{scr}^{v,r} * x + \Delta_{sp}^{v,r}$$

For simplicity, we have not considered the fact that increasing screening frequency would further increase the cases detected by partner notification by a factor. Thus, our counterfactual scenario ignores this relationship. Thus, the decrease in the incidence rate of syphilis predicted by our model by increasing the (routine) screening rate of syphilis might slightly be underestimated by our model.

However, we cannot directly derive $\Delta_{ID}^{v,r}$ as we have limited information on $\Delta_{sp}^{v,r}$. Hence, we derive $\Delta_{ID}^{v,r}$ based on the syphilis notification data to FOPH.[1]
80% of the syphilis cases reported in the year 2017 were found in the primary and secondary stage of syphilis (average time of 3 months). i.e., 80% of the infected MSM in the model were diagnosed with syphilis. The rest 20% of the infected MSM become non-infectious. The rate of becoming non-infectious from infected = 4 (see table 2). By Fellers theorem, the probability of the next event is proportional to the rate. This corresponds to the following equation:

$$\begin{aligned}
\frac{\Delta_{ID}}{\Delta_{IL}} &= \frac{80}{20} \\
\text{or, } \Delta_{ID} &= 4\Delta_{IL} = 4 * 4 \\
\Delta_{ID} &= 16 \\
\text{where,} & \\
\Delta_{ID} &= \text{Rate of becoming diagnosed from infected} \\
\Delta_{IL} &= \text{Rate of becoming non-infectious from infected} = 4
\end{aligned}$$

11 Additional sensitivity analysis

We assessed the robustness of the model by performing additional sensitivity analysis where we sampled 50 sets of fixed parameters from reasonable bounds (table 5) around the point estimates used in the main model using a Latin hypercube sampling algorithm.

Table 5: Bounds of non-free parameters used in sensitivity analysis

Variable	Value used in main model	Chosen bounds	Reason
η^1	0.34	U(0.306, 0.374)	10% uniform bound
η^0	0.91	U(0.865, 0.956)	5% uniform bound
τ	3.167	95% CI (2.721, 3.686)	Derived from SHCS (section 3.3)
$\Delta_{IL}^{v,r}$	4 year^{-1}	U(3.6, 4.4)	10% uniform bound
$\Delta_{LD}^{1,r}$	1 year^{-1}	U(0.9, 1.1)	10% uniform bound
$\Delta_{LD}^{0,r}$	0.5 year^{-1}	U(0.45, 0.55)	10% uniform bound
$\Delta_{ID}^{v,r}$	16 year^{-1}	U(14.4, 17.6)	10% uniform bound
λ	12 year^{-1}	U(10.8, 13.2)	10% uniform bound
κ^1	0.74 year^{-1} in 2006 to 0.45 year^{-1} in 2017	Based on the CI in figure 4	Derived from the SHCS (section 6)
σ^1	0.49 year^{-1} in 2006 to 0.41 year^{-1} in 2017	Based on the CI in figure 4	Derived from the SHCS (section 6)
κ^0	0.54 year^{-1}	-	Model assumption (section 6)
σ^0	1.44 year^{-1} in 2006 to 1.48 year^{-1} in 2017	5% Uniform bounds to figure 5	5% uniform bounds

We fitted the model for each of the 50 fixed parameters sets using the optimization steps described in supplementary section 7. Table 6 summarizes the fitted parameters, goodness of fit, median incidence rate for main model fit and 50 models fit on fixed parameters sets. Figure 19 shows the model fits for the 50 models fit based on the fixed parameters using a Latin hypercube sampling algorithm. Figure 20 shows the corresponding results of counterfactual scenario of increasing the screening frequency of syphilis in MSM with HIV diagnosis and with nsP twice per year instead of annual screening.

Table 6: Fitted parameters for main model fit and re-calibrated models on fixed parameter sets

Parameters	Main model	50 Model fits on sampled parameter sets; Median (IQR)
β_0 (year^{-1})	1.27	1.29 (0.99 - 1.83)

β_1 (year^{-1})	21.88	21.74 (20.25 - 23.01)
θ (year^{-1})	0.0038	0.0038 (0.00377 - 0.00382)
f	0.64	0.59 (0.53 - 0.66)
$\text{inf}_0(0)$	0.38	3.75 (2.71 - 4.90)
$\text{inf}_1(0)$	2.97	2.09 (1.67 - 2.43)
Goodness of fit (SSR)	148.3	159.1 (151.1 - 178.5)
Median IR (per 1000 py)	3.77	3.77 (3.68 - 3.88)

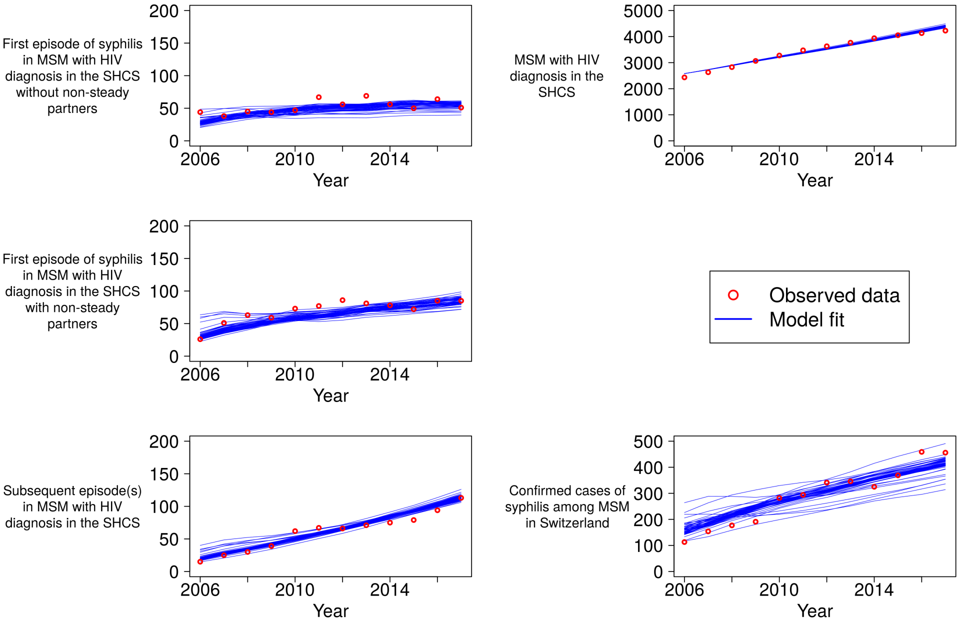

Figure 19: Additional sensitivity analysis: Model fits based on sets of fixed parameters

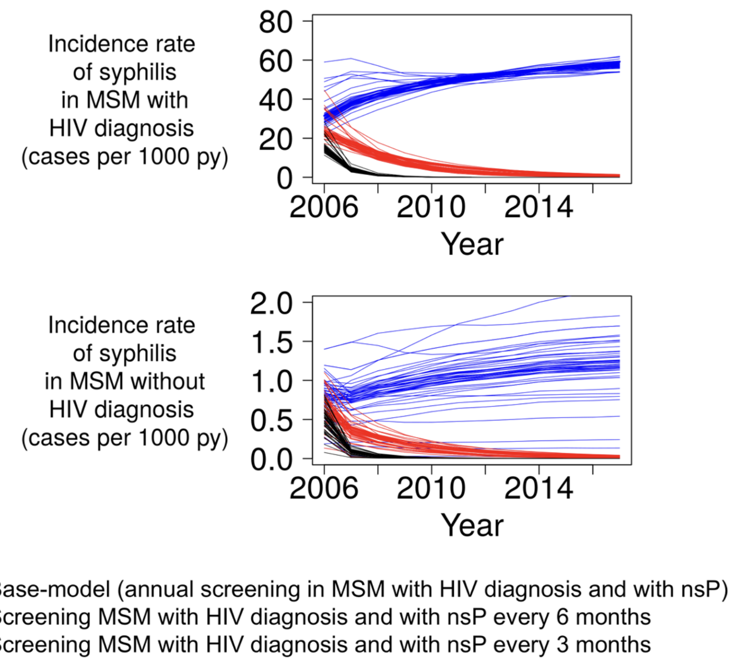

Figure 20: Counterfactual scenario of additional sensitivity analysis: impact of change in screening frequency for syphilis on model fits based on sets of fixed parameters

Thus, our models could reproduce the epidemic of syphilis in Switzerland for the sampled 50 sets of fixed parameters using a Latin hypercube sampling algorithm. Furthermore, increasing the frequency of screening for syphilis among MSM with HIV diagnosis and with non-steady partners from once per year to twice per year during the observation period, we estimated an overall reduction of at least 97.30% in incidence rate of syphilis in 2017 compared to incidence rate obtained by the respective optimized models in 2017. As expected, we observed a high uncertainty in the simulated incidence rate of syphilis in MSM without HIV diagnosis (range: 0.03 to 2.16 cases per 1000 person-years in 2017) due to limited evidence for parameters of MSM without HIV diagnosis. Despite the uncertainty in incidence rate of MSM without HIV diagnosis, increasing the frequency of screening for syphilis among MSM with HIV diagnosis and with non-steady partners from once per year to twice per year during the observation period, we still observed a reduction of at least 97.15% in syphilis incidence in MSM without HIV diagnosis in 2017. These results suggest that our model is quite robust to changes in the chosen fixed parameters of the model.
